# Dietary patterns are associated with obesity in Japanese patients with schizophrenia

**DOI:** 10.1186/1471-244X-14-184

**Published:** 2014-06-20

**Authors:** Norio Sugawara, Norio Yasui-Furukori, Yasushi Sato, Manabu Saito, Hanako Furukori, Taku Nakagami, Masamichi Ishioka, Sunao Kaneko

**Affiliations:** 1 Department of Neuropsychiatry, Hirosaki University School of Medicine, 5 Zaifucho, Hirosaki City, Aomori 036-8562, Japan; 2 Department of Psychiatry, Hirosaki-Aiseikai Hospital, Hirosaki, Japan; 3 Department of Psychiatry, Kuroishi-Akebono Hospital, Kuroishi, Japan; 4 Department of Psychiatry, Odate Municipal General Hospital, Odate, Japan

**Keywords:** Cross-sectional study, Schizophrenia, Dietary patterns, Obesity, Japanese

## Abstract

**Background:**

Obesity among patients with schizophrenia is a growing concern because being overweight is widely regarded as a major risk factor for cardiovascular disease and premature death. Dietary patterns have been suggested as one modifiable factor that may play a role in development of obesity. The objective of this study was to examine the association between dietary patterns and obesity among patients with schizophrenia in Japan.

**Methods:**

We recruited patients (n = 338) aged 44.0 ± 13.2 (mean ± SD) years with a DSM-IV diagnosis of schizophrenia who were admitted to four psychiatric hospitals using a cross-sectional design. Diet was assessed with a validated brief-type self-administered diet history questionnaire (BDHQ). Dietary patterns from 52 predefined food groups were extracted by principal component analysis.

**Results:**

A total of 61 subjects (18.0%) were classified as obese. Three dietary patterns were identified: the healthy dietary pattern, the processed food dietary pattern, and the alcohol and accompanying dietary patterns. After adjusting for age and gender, patients within the high tertile of each healthy dietary pattern (OR = 0.29, 95% CI = 0.13 to 0.62) and processed food dietary pattern (OR = 0.44, 95% CI = 0.22 to 0.89) had a significantly lower risk for obesity compared with low tertile of dietary pattern.

**Conclusions:**

Our findings suggest that dietary patterns, including higher intake of protein, fat, n-3 polyunsaturated fatty acids, n-6 polyunsaturated fatty acids, and vitamins, may be related to a decreased prevalence of obesity within patients with schizophrenia. Future longitudinal research exploring dietary patterns and obesity among patients with schizophrenia is warranted.

## Background

Obesity is a growing concern because being overweight is widely regarded as a major risk factor for metabolic syndrome [1], cardiovascular disease [2], and premature death [3,4]. The prevalence of obesity among patients with schizophrenia is higher [5,6], and the life expectancy of patients with schizophrenia is approximately 15 years shorter for women and 20 years shorter for men, compared to the general population [7]. Although the mechanisms for this weight gain have not been entirely elucidated, dietary factors may be important in the development of obesity.

Diet consists of combinations of foods, and these individual components may have interactive or synergistic effects that make studying dietary factors in isolation difficult [8]. Dietary patterns that represent a combination of foods may be more strongly associated with disease risk than an individual food or nutrient [9-11]. Previous studies have reported that dietary patterns that are high in fruits, vegetables, and fibre might be associated with a reduced risk of obesity [12]. The European Prospective Investigation into Cancer and Nutrition (EPIC) Potsdam cohort found that dietary patterns that are high in fruit, and vegetables and low in high-fat dairy are associated with significantly less weight gain over a 4-year period [13]. In a US study, consuming a diet high in fruit, vegetables, and reduced-fat dairy, and low in meat, fast food, was associated with smaller gains in body mass index and waist circumference [14]. However, no study has investigated associations between dietary patterns and obesity among patients with schizophrenia.

In the present study, we examined the association between dietary patterns and obesity among patients with schizophrenia living in Japan. We hypothesised that dietary patterns consisting of high intake of vegetables and fish would be associated with a decreased risk of obesity.

## Methods

### Participants

This study was conducted between June 2011 and August 2011. The subjects included 338 outpatients (170 males and 168 females) who were diagnosed with either schizophrenia or schizoaffective disorder based on Diagnostic and Statistical Manual of Mental Disorders, fourth edition (DSM-IV) criteria at four psychiatric hospitals in Japan. The diagnoses were determined based upon medical records. The data collection for this study was approved by the Ethics Committee of the Hirosaki University School of Medicine (2011–039), and all subjects provided written informed consent before participating in this study.

### Procedure

Subjects’ demographic data (age and sex) were obtained from their medical records. Each subject’s height and weight were measured, and the body mass index (BMI) was calculated as the individual’s body mass divided by the square of their height (kg/m^2^). Participants were classified as being obese if their BMI was 30 or above.

Dietary habits during the preceding month were assessed using a validated brief self-administered diet history questionnaire (BDHQ) that contained questions about the consumption frequency of 56 foods and beverages and 9 dishes that are commonly consumed in the general Japanese population [15]. Dietary intake of energy and selected nutrients were estimated using an ad hoc computer algorithm for the 56 foods and beverages of the BDHQ and the Standard Tables of Food Composition in Japan [16,17].

### Statistical analyses

The data were analysed using the PASW Statistics PC software for Windows, Version 18.0.0 (SPSS Inc., Chicago, IL, USA) and R 2.10.1. was used for the Cochran-Armitage trend test only [18]. We derived dietary patterns through a principal component analysis of energy-adjusted intake that used a density method for the 52 included food and beverage items (4 items that overlapped with others) [19]. We used eigen values, the scree test [20], and the interpretability of the factors to determine how many factors to retain. The factors each had eigen values greater than one. The scree plots dropped substantially after the third factor (from 2.39 to 2.05) and remained similar after the fifth factor (2.05 for the fourth and 1.94 for the fifth factor); thus, we retained three factors. The factor scores for each dietary pattern and for each individual were calculated by summing the food item intakes weighted by their factor loadings. The factor scores were categorised into tertiles.

Either Student’s unpaired *t*-test (for the continuous variables) or the chi-square test (for categorical variables) was used to compare participants with and without obesity. Trend associations across the tertile categories of each dietary pattern were assessed using the Cochran-Armitage trend test for categorical variables and linear regression analysis for continuous variables, with ordinal values from 0 to 2 being assigned to the tertile categories of each dietary pattern. A logistic regression analysis was used to assess the relationships between dietary patterns and obesity. The model was adjusted for age and gender. A result with p < 0.05 was considered to be significant.

## Results

### Characteristics of participants with and without obesity

The subjects were divided into two groups according to their BMI (subjects with obesity BMI ≥ 30, n = 61; subjects without obesity BMI < 30, n = 277). The characteristics of both subject groups are listed in Table 1. Subjects with obesity were significantly younger; had higher BMIs; and had lower intake of protein, dietary fibre, folate, pyridoxine, and ascorbic acid. No other differences were observed.

**Table 1:** Characteristics of subjects with and without obesity

	**Subjects with obesity**^**a**^	**Subjects without obesity**^**b**^	**p value**^**c**^
Number of subjects	61	277	
Age	40.7 ± 12.1	44.7 ± 13.3	<0.05
BMI	34.0 ± 3.4	23.6 ± 3.3	<0.001
Male (%)	29 (47.5)	141 (50.9)	0.635
Dietary intake			
Energy (kcal)	1807 ± 1036	1775 ± 778	0.780
Protein (g/1000 kcal)	34.7 ± 9.4	37.1 ± 7.7	<0.05
Carbohydrate (g/1000 kcal)	142 ± 27	141 ± 21	0.752
Fat (g/1000 kcal)	27.1 ± 8.1	28.0 ± 7.1	0.392
Dietary fibre (g/1000 kcal)	5.18 ± 2.38	6.20 ± 2.60	<0.01
Alcohol (g/1000 kcal)	4.35 ± 10.06	2.89 ± 8.14	0.294
n-3 polyunsaturated fatty acids (g/1000 kcal)	1.30 ± 0.54	1.42 ± 0.49	0.091
n-6 polyunsaturated fatty acids (g/1000 kcal)	5.19 ± 1.84	5.47 ± 1.53	0.218
Folate (μg/1000 kcal)	139 ± 74	162 ± 82	<0.05
Riboflavin (Vitamin B2) (mg/1000 kcal)	0.66 ± 0.25	0.67 ± 0.21	0.559
Pyridoxine (Vitamin B6) (mg/1000 kcal)	0.55 ± 0.19	0.61 ± 0.19	<0.05
Cobalamin (Vitamin B12) (μg/1000 kcal)	4.40 ± 2.80	4.95 ± 2.76	0.165
Ascorbic acid (Vitamin C) (mg/1000 kcal)	41.5 ± 30.3	51.8 ± 33.0	<0.05

*Abbreviation*: *BMI* body mass index.

^a^Subjects with BMI ≥ 30.

^b^Subjects with BMI < 30.

^c^For continuous variables, non-paired t-tests were used; for categorical variables, chi-square tests were used.

### Dietary patterns identified by principal component analyses

We identified three dietary patterns by principal component analysis (Table 2). The first factor, which loaded heavily on vegetables, seaweeds, tofu, fruits, and fish, was labelled the “healthy” dietary pattern. The second factor, which had high loadings for ham/sausage/bacon, noodles, pasta, fruit, and confectioneries, was labelled the “processed food” dietary pattern. The third factor was characterised by high intake of alcoholic beverages, noodles, and liver; thus, it was termed the “alcohol and accompanying” dietary pattern. These three dietary patterns accounted for 10.2%, 4.9%, and 4.6%, respectively, of the variance in food intakes and explained 19.7% of the variance.

**Table 2:** **Factor-loading matrix for major dietary patterns identified by principal component analysis**^**a**^

	**Healthy dietary pattern**	**Processed food pattern**	**Alcohol and accompanying dietary pattern**
Reduced fat milk and yogurt	0.197	-	-
Milk and yogurt	-	0.241	−0.169
Chicken	-	0.164	-
Ham/sausage/bacon	-	0.332	-
Liver	-	0.280	0.285
Squid/octopus/shrimp/shellfish	-	0.272	-
Small fish with bones	0.274	-	-
Canned tuna	-	0.267	-
Dried fish/salted fish	-	0.174	-
Oily fish	0.209	0.154	-
Lean fish	0.168	-	-
Tofu/atsuage^b^	0.399	0.157	-
Natto^c^	0.369	-	-
Potatoes	0.285	0.153	-
Pickled green leafy vegetables	0.484	-	-
Other pickled vegetables	0.238	-	0.164
Lettuces/cabbage (raw)	0.751	-	-
Green leafy vegetables	0.778	−0.207	-
Cabbage/Chinese cabbage	0.626	-	-
Carrots/pumpkin	0.610	-	-
Japanese radish/turnip	0.574	-	0.256
Other root vegetables	0.618	0.179	-
Tomatoes	0.510	−0.249	−0.210
Mushrooms	0.664	−0.150	-
Seaweeds	0.650	−0.269	-
Western-type confectioneries	-	0.323	−0.480
Japanese-type confectioneries	-	0.255	−0.404
Rice crackers/rice cake/okonomiyaki	-	0.236	−0.331
Ice cream	−0.158	-	−0.174
Citrus fruit	0.177	0.327	−0.189
Persimmons/strawberries/kiwifruit	0.344	0.281	-
Other fruit	0.166	0.276	−0.251
Mayonnaise	0.413	-	-
Bread	-	0.238	−0.307
Buckwheat noodles	-	0.254	0.278
Japanese wheat noodles	0.179	0.347	0.408
Chinese noodles	−0.177	0.178	0.362
Pasta	-	0.221	0.318
Green tea	0.193	-	-
Black tea/oolong tea	-	0.225	0.156
Cola drink/soft drink	−0.271	-	-
100% fruit and vegetable juice	-	0.171	-
Rice	−0.195	−0.762	-
Miso soup	-	−0.340	-
Sake	-	-	0.270
Beer	-	-	0.460
Shochu	-	-	0.408
Whisky	-	-	0.356
Wine	-	0.150	0.321

^a^Factor loading less than ± 0.15 represented by a dash for simplicity. Omitted in the table were food items with factor loadings less than ± 0.15 for all dietary patterns (Pork/beef, Egg, and Coffee).

^b^Deep-fried tofu.

^c^Fermented soybeans.

### Characteristics according to tertile categories of dietary pattern scores

Table 3 shows the characteristics according to tertiles of the dietary pattern scores. The subjects with higher scores for the healthy dietary pattern were more likely to have lower BMIs and were less likely to be males. The healthy pattern was positively associated with intake of protein, fat, dietary fibre, n-3 polyunsaturated fatty acids (PUFA), n-6 PUFA, folate, riboflavin, pyridoxine, cobalamin, and ascorbic acid and was inversely associated with the intake of carbohydrates.

**Table 3:** Characteristics according to tertile categories of dietary pattern scores

		**Healthy dietary pattern**				**Processed food pattern**				**Alcohol and accompanying dietary pattern**			
	**Total**	**Low tertile**	**Middle tertile**	**High tertile**	**Trend p**^**a**^	**Low tertile**	**Middle tertile**	**High tertile**	**Trend p**^**a**^	**Low tertile**	**Middle tertile**	**High tertile**	**Trend p**^**a**^
Number of subjects	338	112	113	113		113	112	113		112	113	113	
Age	44.0 ± 13.2	42.9 ± 12.1	43.7 ± 14.0	45.3 ± 13.4	0.181	45.2 ± 13.5	43.5 ± 12.2	43.2 ± 13.9	0.253	43.3 ± 13.5	45.4 ± 13.7	43.2 ± 12.2	0.980
BMI	25.5 ± 5.2	26.7 ± 5.7	25.6 ± 5.3	24.2 ± 4.2	<0.001	26.3 ± 5.0	25.3 ± 5.3	24.8 ± 5.3	<0.05	25.2 ± 5.0	25.1 ± 4.7	26.0 ± 5.8	0.294
Male (%)	170 (50.3)	71 (63.4)	60 (53.1)	39 (34.5)	<0.001	63 (55.8)	59 (52.7)	48 (42.5)	<0.05	47 (42.0)	48 (42.5)	75 (66.4)	<0.001
Dietary intake													
Energy (kcal)	1781 ± 829	1679 ± 872	1877 ± 822	1788 ± 785	0.315	1656 ± 591	1670 ± 626	2014 ± 1116	<0.01	1810 ± 657	1710 ± 788	1823 = 1005	0.904
Protein (g/l000 kcal)	36.6 ± 8.1	31.7 ± 6.9	36.8 ± 7.7	41.4 ± 6.4	<0.001	33.7 ± 7.3	36.6 ± 7.4	39.6 ± 8.3	<0.001	36.5 ± 7.2	37.3 ± 7.7	36.1 ± 9.1	0.721
Carbohydrate (g/l000kcal)	141 ± 22	148 ± 24	139 ± 21	136 ± 19	<0001	154 ± 18	138 ± 21	131 ± 21	<0001	140 ± 19	146 ± 20	138 ± 26	0.408
Fat (g/1000 kcal)	27.9 ± 7.3	25.6 ± 8.0	28.6 ± 6.8	29.4 ± 6.6	<0.001	23.5 ± 6.0	28.2 ± 6.8	31.9 ± 6.6	<0.001	30.9 ± 6.8	27.2 ± 6.5	25.6 ± 7.6	<0.001
Dietary fibre (g/l000 kcal)	6.02 ± 2.59	4.11 ± 1.23	5.49 ± 1.00	8.44 ± 2.80	<0.001	5.74 ± 3.08	5.92 ± 2.37	6.38 ± 2.21	0.066	5.96 ± 2.84	6.11 ± 2.47	5.98 = 2.47	0.948
Alcohol (g/l000kcal)	3.16 ± 8.52	4.22 ± 10.72	3.14 ± 8.54	2.12 ± 5.42	0.065	2.49 ± 6.06	4.31 ± 11.38	2.68 ± 7.19	0.871	0.51 ± 1.49	0.80 ± 2.18	8.13 ± 13.19	<0.001
n-3 polyunsaturated fatty acids (g/l000	1.40 ± 0.50	1.08 ± 0.41	1.43 ± 0.41	1.68 ± 0.49	<0.001	1.22 ± 0.42	1.43 ± 0.50	1.54 ± 0.52	<0001	1.48 ± 0.52	1.37 ± 0.45	1.35 ± 0.52	<0.05
n-6 polyunsaturated fatty acids (g/l000	5.42 ± 1.59	4.83 ± 1.82	5.56 ± 1.32	5.85 ± 1.44	<0.001	4.80 ± 1.31	5.54 ± 1.74	5.91 ± 1.50	<0.001	5.60 ± 1.57	5.34 ± 1.32	5.32 ± 1.84	0.191
Folate (μg/l000kcaI)	158 ± 81	99 ± 34	147 ± 32	228 ± 96	<0.001	150 ± 102	153 ± 69	172 ± 67	<0.05	157 ± 94	157 ± 76	161 ± 74	0.753
Riboflavin (Vitamin B2) (mg/1000 kcal)	0.67 ± 0.22	0.55 ± 0.18	0.68 ± 0.19	0.79 ± 0.21	<0.001	0.60 ± 0.23	0.66 ± 021	0.75 ± 0.18	<0.001	0.69 ± 0.22	0.68 ± 0.22	0.65 ± 0.21	0.145
Pyridoxine (Vitamin B6) (mg/1000 kcal)	0.60 ± 0.19	0.45 ± 0.14	0.59 ± 0.12	0.76 ± 0.16	<0.001	0.54 ± 0.19	0.60 ± 0.17	0.67 ± 0.18	<0.001	0.60 ± 0.18	0.60 ± 0.19	0.61 ± 0.20	0.879
Cobalamin (Vitamin Bl2) (μg/l000 kcal)	4.85 ± 2.78	3.47 ± 2.42	5.03 ± 2.63	6.03 ± 2.67	<0.001	3.78 ± 1.86	4.95 ± 2.63	5.81 ± 3.28	<0.001	4.89 ± 2.56	4.66 ± 2.74	4.99 ± 3.02	0.797
Ascorbic acid (Vitamin C) (mg/l000 kcal)	49.9 ± 32.7	29.2 ± 20.2	44.6 ± 19.2	75.8 ± 36.2	<0.001	42.3 ± 35.1	46.6 ± 27.7	60.9 ± 32.2	<0.001	51.4 ± 35.4	49.0 ± 30.6	49.4 ± 32.1	0.649

*Abbreviation*: *BMI* body mass index.

^a^Based on the Cochram-Armitage trend test for categorical variables and linear regression anlysis for continuous variables; ordinal numbers 0–2 were assigned to the tertile categories of each dietary pattern.

The subjects with higher scores for the processed food dietary pattern were more likely to have lower BMIs and less likely to be males. The processed food pattern was positively associated with intake of total energy, protein, fat, n-3 PUFA, n-6 PUFA, folate, riboflavin, pyridoxine, cobalamin, and ascorbic acid and was inversely associated with the intake of carbohydrates. The subjects with higher scores for the alcohol and accompanying dietary pattern were more likely to have higher BMIs and to be males. The alcohol and accompanying pattern was positively associated with alcohol consumption and was inversely associated with intake of fat and n-3 PUFA.

### Odds ratio (OR) and 95% confidence intervals (CIs) for obesity according to tertiles of dietary pattern scores

The odds ratios for obesity according to the tertile categories for each dietary pattern score are shown in Table 4. After adjusting for age and gender, patients within the high tertile of each healthy dietary pattern (OR = 0.29, 95% CI = 0.13 to 0.62) and processed food dietary pattern (OR = 0.44, 95% CI = 0.22 to 0.89) had a significantly lower risk for obesity compared with low tertile of dietary pattern. In the same condition, subjects in the high tertile for the alcohol and accompanying dietary pattern showed a tendency (OR = 1.80, 95% CI = 0.90 to 3.59) toward a higher risk of obesity compared with the low tertile.

**Table 4:** Odds ratio and 95% CIs for obesity according to tertiles of dietary pattern scores

	**No. of cases**	**Crude odds ratio**	**p value**	**Adjusted odds ratio**	**p value**
Healthy dietary pattern					
Low tertile	29	Reference		Reference	
Middle tertile	21	0.65 (0.35-1.23)	0.189	0.63 (0.33-1.20)	0.160
High tertile	11	0.31 (0.15-0.66)	<0.01	0.29 (0.13-0.62)	<0.01
Processed food pattern					
Low tertile	27	Reference		Reference	
Middle tertile	19	0.65 (0.34-1.25)	0.199	0.62 (0.32-1.20)	0.153
High tertile	15	0.49 (0.24-0.98)	<0.05	0.44 (0.22-0.89)	<0.05
Alcohol and accompanying dietary pattern					
Low tertile	18	Reference		Reference	
Middle tertile	16	0.86 (0.42-1.79)	0.689	0.91 (0.44-1.91)	0.804
High tertile	27	1.64 (0.84-3.19)	0.145	1.80 (0.90-3.59)	0.095

*Abbreviation*: *CI* confidence interval.

Logistic regression model was adjusted for age and gender.

## Discussion

Patients with schizophrenia have poorer diet quality (e.g., consume less dietary fibre and vitamins) than the general population [21,22], but few studies of the association between diet quality and obesity have been conducted in schizophrenic populations. Our results from a cross-sectional study are the first evidence to suggest that a healthier dietary pattern may be instrumental in reducing obesity in patients with schizophrenia.

There have been two main types of approach used to extract dietary patterns. The first approach aims to calculate a graded score or index based on recommended diets or dietary guidelines [23]. This technique was called an *a priori* or hypothesis-oriented approach. The weakness of hypothesis-oriented approaches is that they focus on selected aspects of diet and do not consider the correlation structure of food and nutrient intakes. Consequently, such scores do not reflect the overall effect of diet in general but only the formal sum of not-adjusted single effects. The second approach, so called an *a posteriori* or exploratory approach, extracts dietary patterns from the data at hand. This approach ignores prior knowledge completely. Statistical exploratory methods that accomplish pattern extraction are principal component analysis and factor analysis, which are widely applied in nutritional epidemiology. Applied to food intake data, exploratory approaches aim to explain the total variation in intake of many foods or food groups.

We identified three major dietary patterns through principal component analysis: the healthy dietary pattern, the processed food pattern, and the alcohol and accompanying pattern. We hypothesised that dietary patterns consisting of a high intake of vegetables and fish would be associated with a decreased risk of obesity. In the present study, we found that subjects who consumed the healthy dietary pattern, characterised by a high intake of vegetables, seaweeds, tofu, fruits, and fish, showed significantly a lower risk for obesity. The processed food dietary pattern, characterised by a high intake of ham/sausage/bacon, noodles, pasta, fruit, and confectioneries, was also significantly associated with a lower risk of obesity. Because these two dietary patterns had different trends in total energy intake, just total energy intake alone does not uniquely cause obesity in schizophrenia. Although it remains unclear which components or nutritive factors might mediate the association between the observed dietary patterns and obesity among patients with schizophrenia, nutrients that are commonly included among these two dietary patterns, such as higher intake of protein, fat, n-3 PUFA, n-6 PUFA, and vitamins, might affect our results. In addition, a higher intake of these two dietary patterns might be associated with socioeconomic status (education, family income) and life style (smoking, sleeping duration) [24-27].

The prevalence of obesity was markedly different in Japan as compared with Western countries [28-30] in general population. In contrast to previous results conducted among patients with schizophrenia in Western countries [31,32], we found that only 18% of our participants were obese. Differences of lifestyle and ethnicity between Japan and Western countries might affect the lower prevalence of obesity in our study.

The current study has several limitations. First, the cross-sectional nature of the study does not allow for causal assumptions about dietary patterns and the onset of obesity. Future longitudinal studies are needed to investigate these associations. Second, the dietary data were obtained using the BDHQ. Although the validity and reliability of our dietary questionnaire have been previously evaluated [15,18], potential misclassification of dietary patterns may have affected our results. Third, several potential confounding factors, such as physical activity levels, socioeconomic status, life style, severity of schizophrenia, and antipsychotic medications, were not assessed in our study. Future studies adjusted for the above confounds are needed. Fourth, because all of the participants were volunteers who were interested in their health, they may not be representative of typical subjects with schizophrenia. Thus, patients who were not in the study may have different symptoms. This ‘selection bias’ must also be considered in studies of community populations. Finally, because our sample size was relatively small, we could not completely rule out beta error as the cause of the lack of a significant association between alcohol and accompanying dietary patterns and obesity.

## Conclusions

Our findings suggest that dietary patterns with higher intakes of protein, fat, n-3 PUFA, n-6 PUFA, and vitamins may be related to a decreased prevalence of obesity. Intervention programs to change dietary patterns among patients with schizophrenia might be useful for treating obesity. Future research exploring dietary patterns and obesity among patients with schizophrenia is warranted, especially studies employing a longitudinal design.

## Competing interests

The authors declare that they have no competing interests.

## Authors’ contributions

NS conceived the study, designed the study, conducted the statistical analysis, interpreted the data and wrote the initial draft of the manuscript. SK had full access to all of the data in the study and takes responsibility for the integrity of the data and the accuracy of the data analysis. NYF contributed to study design and assisted in drafting the manuscript. YS and HF completed initial survey construction, recruitment of participants. MS, TN, and MI participated in the data collection, and the interpretation of the results. All authors have approved the manuscript.

## Pre-publication history

The pre-publication history for this paper can be accessed here:

http://www.biomedcentral.com/1471-244X/14/184/prepub
